# Ramsay Hunt syndrome: characteristics and patient self-assessed long-term facial palsy outcome

**DOI:** 10.1007/s00405-020-05817-y

**Published:** 2020-01-25

**Authors:** Mervi Kanerva, Sanna Jones, Anne Pitkaranta

**Affiliations:** 1 Department of Otorhinolaryngology-Head and Neck Surgery, Helsinki University Hospital and University of Helsinki, PO Box 263, 00029 HUS Helsinki, Finland; 2 Terveystalo Occupational Healthcare Services, Leppavaara and Espoontori, Espoo, Finland

**Keywords:** Herpes zoster oticus, Facial paralysis, Varicella–Zoster virus, House–Brackmann, Sunnybrook, Facial grading, Facial sequelae

## Abstract

**Purpose:**

To explore the characteristics, medical treatments, and long-term facial palsy outcome in Ramsay Hunt syndrome.

**Methods:**

Patient questionnaire including self-assessment of long-term facial palsy outcome and retrospective chart review. Initial facial palsy grade was compared to self-assessed or patient record stated palsy outcome. Occurrence of different characteristics (blisters, hearing loss, vertigo, etc.) of the syndrome were assessed.

**Results:**

Altogether 120 patients were included of which 81 answered the questionnaire. All but one patient had received virus medication (aciclovir, valaciclovir), and half received simultaneous corticosteroids. If the medication was started within 72 h of Ramsay Hunt diagnosis, facial palsy recovered totally or with only slight sequelae in over 80% of the patients. Only a minority of the patients experienced varicella blisters simultaneously with facial palsy, blisters more often preceded or followed the palsy. Approximately 20% of the patients had their blisters in hidden places in the ear canal or mouth.

**Conclusions:**

The long-term outcome of facial palsy in medically treated Ramsay Hunt syndrome was approaching the outcome of Bell’s palsy. It is crucial to ask and inform the patient about the blisters and look for them since, more often than not, the blisters precede or follow the palsy and can be in areas not easily seen.

## Introduction

Ramsay Hunt syndrome (RHS) consists of peripheral facial palsy (FP) of the VII cranial nerve (CN) and herpes blisters in the head and neck area. All nerves that communicate with the facial nerve can be involved e.g. CNs V, VIII, IX, and X and cervical nerves C2, C3, and C4 [1]. Hearing loss, tinnitus, and vertigo frequently accompany facial palsy [2]. RHS is caused by reactivation of latent varicella-zoster virus (VZV) [2, 3]. Even though cranial polyneuropathy including many additional CNs (e.g. III, XI, XII) was not included in the original definition of RHS, investigators have reported such patients as rare cases of RHS [1, 4, 5].

There are no prospective studies on RHS, specifically. Peitersen [6] studied idiopathic peripheral FP and gathered all cases throughout 25 years in Copenhagen, Denmark. In this group of 2570 FP patients, there were 116 cases of RHS. The annual incidence was 2.2/100,000; which was approximately 4.5% of all FPs. Estimates of incidence based on mostly retrospective studies of individual centers have ranged 4–12% of all FPs and around 5/100,000/year [7, 8].

The clinical picture of RHS includes three possibilities regarding the timing of the relationship between FP and blisters. If the blisters occur before or simultaneously with the palsy, RHS diagnosis is usually easy. Sometimes the blisters are not very noticeable and can be overlooked by the inspector, if RHS is not considered in patients experiencing FP. Similarly, in cases where FP presents first, and blisters later, clinical RHS diagnosis is impossible at the time of palsy onset.

Recognizing RHS has become more important with the widely accepted practice of treating these patients with antiviral agents along with corticosteroids even though consensus is missing [9–11]. Comparing recovery results from the time before and after the use of antiviral treatments and knowing that shingles patients benefit from medical therapy [12, 13], recommendations have been published for the combined use of antivirals and corticosteroids [11, 14]. No prospective medical studies likely will exist that compare different medical treatments to no medications at all in RHS, but we can study the patients and how they have recovered in order to draw some suggestive conclusions about the treatment results.

The aim of this study was to investigate the characteristics and treatment of RHS and to determine the long-term outcome of FP in RHS patients using patient charts retrospectively and one-time patient questionnaire.

## Materials and methods

A computer search of patient records for RHS from 1/1996 to 1/2013 was performed in Helsinki University Central Hospital, Department of Otorhinolaryngology-Head and Neck Surgery. Patients with peripheral FP and a history or recognition of blisters in the head and neck area within 1 month prior, concomitantly with palsy onset, or 1 month following the palsy onset, were included in the study. Some patients had to be removed from the study, since the history of the blisters was indefinite, and one patient because of a malignancy behind the symptoms. The time, when RHS diagnosis was possible to make, was considered RHS onset.

Besides using the patient records for the data collection, the patients were sent a questionnaire asking about their RHS symptoms and how they had recovered, and were asked to grade their FP outcome.

FP grading methods in patient records varied and both House–Brackmann (HB) [15] and Sunnybrook (SB) [16] grading scales had been used. For study purposes, SB grades were converted to HB grades according to Conversion Table [17].

We used SPSS (Armonk, NY: IBM Corp.) versions 22–24 for statistical analyses. We used descriptive statistics to summarize the frequencies, proportions, medians, and ranges. Fischer’s Exact Test was used to calculate the significances.

## Results

### Patients

Inclusion criteria were met by 120 patients: 71 women and 49 men. At the time of the study, 8 of the 120 patients had died and 4 could not be reached. The remaining 108 patients were sent a questionnaire about their RHS. Of these, 81 answered the questionnaire (52 women, 29 men). The caregiver of an additional three patients informed that due to the patients’ illnesses, they were no longer capable of answering the questionnaire.

Of the 120 patients, 59 experienced FP on the right, and 61 on the left. Of the 81 patients answering the questionnaire, 40 experienced FP on the right, and 41 on the left.

Age at time of RHS onset for the 120 patients varied from 6.9 to 94.7 years. The median age for women was 56.4 years (range 12.2–94.7), for men 48.8 years (range 6.9–84.3). Patients answering the questionnaire were aged 6.9–88.2 years. The median age was 52.3 years (range 2.2–88.2) for women, and 49.1 years (range 6.9–84.3) for men. Five of these patients were 16 years or younger at RHS onset.

The median follow-up time for the 81 patients answering the questionnaire was 6.6 years (range 1–17.1). For those failing to answer the questionnaire, the data were solely collected from the patient records: FP of 26 patients had recovered to total or near total in a median of 3.4 months (range 0.6–12.1) and no additional follow-up times were scheduled. For the remaining 13 (of the 120 patients), 4 were lost in follow-up after their initial visit and 9 patients had a follow-up median of 7.2 months (range 1.2–12.1).

### Medical treatment

At RHS onset, all but one of the 120 patients were prescribed either intravenous or oral antiviral medication (aciclovir or valaciclovir) (Table 1). Of the prescription-receiving patients, 57 (48%) were also prescribed concomitant corticosteroids (prednisolon or methylprednisolon). One patient receiving both aciclovir and prednisolone prescription admitted not taking either of the medicines and was placed in the “no-medication” group for the outcome results in this study.

**Table 1:** Long-term outcome of Ramsay Hunt syndrome facial palsy combined from patient records and patient’s self-assessment questionnaire and compared to initial facial palsy grade

Medication at Ramsay Hunt syndrome onset	Number of patients (total = 120)	Medication onset time from Ramsay Hunt diagnose/patient number Initial HB grade^a^ –› outcome^b^/patient number			
		≤ 24 h	≤ 48 h	≤ 72 h	> 72 h
Valaciclovir orally 1 g × 3 × 7	18	15 HB II –› 1/3 HB III –› 1/1 HB VI –› 2/1 HB ? –› 1/3 HB ? –› 2/6 HB ? –› 3/1		1 HB ? –› 1/1	2 HB II –›1/1 HB ? –› 2/1
Valaciclovir orally, dosing unclear	3	3 HB V –›1/1 HB V –› 2/1 HB ? –› 1/1			
Valaciclovir orally 1 g × 3 × 7 + corticosteroid^c^	41	37 HB II –› 1/1 HB III –› 1/2 HB III –› 4/1 HB IV –› 1/4 HB IV –› 2/2 HB IV –›?/1 HB V –›1/1 HB V –› 2/1 HB V –› 3/1 HB VI –› 1/3 HB VI –› 3/3 HB VI –› ?/1 HB ? –› 1/7 HB ? –› 2/7 HB ? –› 3/2	2 HB II –› 1/1 HB V –› 1/1	1 HB V –› 1/1	1 HB V –› 3/1
Aciclovir orally 800 mg × 5 × 7	9	7 HB III –› 2/1 HB III –› ?/1 HB IV –› 2/1 HB V –›1/1 HB ? –› 1/3			2 HB VI –› 2/1 HB ? –› 1/1
Aciclovir orally 800 mg × 5 × 7 + corticosteroid^c^	3	3 HB II –› 2/1 HB VI –› 3/1 HB ? –› 1/1			
Aciclovir orally, dosing under 800 mg × 5 × 7 or unclear	9	7 HB IV –› 1/1 HB ? –› 1/4 HB ? –›2/2	1 HB ? –› 3/1		1 HB ? –›?/1
Aciclovir intra venous under 10 mg/kg/day × 3 or unclear	3	3 HB II –› 1/1 HB IV –› 1/1 HB ?–› 4/1			
Aciclovir orally under 800 mg × 5 × 7 or intra venous under 10 mg/kg/day × 3 + corticosteroid^c^	3	3 HB II –› 1/1 HB V –› 2/1 HB ? –›1/1			
Aciclovir intra venous minimum 10 mg/kg/day × 3	19	14 HB II –›1/1 HB III –›1/1 HB V –› 1/1 HB V –› 2/1 HB V –› 3/1 HB VI –› 2/1 HB VI –› 3/1 HB ? –› 1/3 HB ? –› 2/2 HB ? –› 4/1 HB ? –›?/1	2 HB ? –› 1/1 HB ? –› 1/1		3 HB II –› 3/1 HB II –›1/1 HB VI –› 4/1
Aciclovir intra venous minimum10 mg/kg/day × 3 + corticosteroid^c^	10	7 HB II –› 2/1 HB IV –› 1/1 HB V –› 1/2 HB V –› 3/1 HB VI –› 4/1 HB ? –› 2/1		1 HB V –› 2/1	2 HB ? –› 3/1 HB ? –›1/1
No medication	2				2 HB ? –› 2/1 HB ? –› 3/1

Patients are grouped by the medication type and medication start time at syndrome onset

^a^Initial facial palsy grade was available from 62 patient records, graded by House-Brackmann scale (HB) or Sunnybrook scale, latter converted here to HB grades

^b^Facial palsy outcome: 1—totally recovered; 2—slight sequelae; 3—obvious sequelae; 4—severe sequelae

^c^Corticosteroid usually Prednisolon 60 mg/day with tapering dosing 10 mg/day after 5 days for 10 days or Medrol 32 mg or 64 mg/day for 7–10 days

Of the 120 patients, 104 (87%) received their medication within 48 h, an additional 3 (2%) patients within 72 h, 12 (10%) patients over 72 h from the onset of RHS (one not consuming the medication), and 1 (1%) received no medication at all (Table 1).

### Blisters

From patient charts, the location of VZV blisters could be identified for 119 patients. The most frequent area to be affected was the ear. Outer ear, with or without ear canal involvement, was the sole place for blisters in 62 patients (52%). In addition, in 7 patients (6%) only the ear canal or the ear drum were infected, with no outside visible blisters. Taken together, the ear by itself counted for 58% of the cases. Ear was additionally involved as follows: ear and mouth 4 patients (3%); ear, mouth, and additional area 3 patients (2%); and ear and additional area (apart from mouth) 15 patients (13%). Thus, the ear was one of the areas affected in 91 patients (76%). Mouth cavity was solely affected in 14 patients (12%) and in addition to prementioned times with ear, in 2 patients (2%) mouth was affected with other area facial blisters. Face, neck, and shoulders without ear or mouth involvement were affected in 12 patients (10%).

VZV blisters preceded FP in 58 patients (48%), were concomitant in 19 patients (16%), and followed the palsy onset in 43 patients (36%).

From 58 patients’ charts, the time difference between the blisters appearing before FP could be assessed: 1–2 days prior in 15 cases (26%), 3–7 days prior in 25 cases (43%), 8–14 days prior in 13 cases (22%), and over 14 (≤ 30) days prior in 5 cases (9%).

The information about the time difference between FP preceeding blisters could be obtained from 43 patient charts: 1–2 days prior in 20 cases (46%), 3–7 days prior in 17 cases (40%), 8–14 days prior in 3 cases (7%), and over 14 (≤ 30) days prior in 3 cases (7%).

### Hearing loss

An audiogram had been taken from 103 patients; In 54 (52%) there was an acute hearing loss linked to the RHS. In 8 of these cases, there was a mention that the hearing loss was mild (the audiogram itself was not obtainable). No comprehensive audiometry testing could be found at the end of the follow-up time for all the study patients. Of those 81 patients answering the questionnaire, 18 (22%) answered that they were left with a hearing loss after the illness, 7 (9%) were not sure, and 56 (69%) answered they had no hearing loss because of RHS.

### Other symptoms

From the patient charts (*n* = 120), mentions of accompaning symptoms could be gathered from 110 patients: symptoms around the affected ear 86 (78%) (ache, fullness e.g.), ache in some other area 24 (22%), vertigo/dizziness 34 (31%), and additional mentions of nausea, vomiting, tinnitus, and hyperacusia.

Of the 81 patients answering the questionnaire, 73 commented on the symptoms from the acute period of RHS onset: ache around the affected ear 56 (77%), vertigo/dizziness 37 (51%), hearing loss 33 (45%), and dryness of the affected eye 31 (42%). Other frequently mentioned symptoms were nausea, tinnitus, dysgeusia, vomiting, and dryness of the mouth. At the questionnaire’s open area for extra reporting “other areas of pain” was mentioned frequently. For the questions about persistent symptoms after minimum of 1 year recovery time 26 (32%) mentioned having persistent vertigo or dizziness because of the RHS, 5 (6%) patients were not sure, and 50 (62%) experienced no balance disturbances. Tinnitus was experienced by 24 (30%) patients, dry mouth by 21 (26%), facial numbness by 21 (26%), and excessive tearing by 23 (28%).

### Immunocompromised patients

Altogether 12 (10%) of the 120 patients either had an illness or took medication that made them immunocompromised and could have predisposed them to RHS.

### FP or RHS history

Amongst those 81 patients answering the questionnaire, 7 (9%) had a relative who had experienced FP. No one had a relative with RHS history. There was one patient with previous FP from which she was totally cured. Two patients had suspected a new FP after the RHS, but there were no patient charts for review of the incidence and the symptoms had disappeared in a day or two. There were no patients with recurrent RHS.

### FP recovery from patient questionnaire

Table 2 shows the FP outcome of patient self-assessment from the questionnaire. There was no statistical difference in outcome, whether the patient received “gold standard” shingles virus medication or whether the amount of the medication was unclear (Tables 2, 3, 4). Similarly, there was no statistical difference whether the medication included corticosteroids or not. The outcome stayed the same as long as the medication was started within 72 h of RHS onset with 64 (84%) of the patients totally cured or left only with slight sequelae (Tables 2, 3, 4). In total paralysis (HB VI) the FP outcome to total or good recovery with medication within 72 h was achieved by 3 of the 7 patients (43%) (Tables 3, 4). Already in HB grade V, the recovery to that same grade was achieved by 5 of 7 and 8 of 10 patients (70–80%) (Tables 3, 4).

**Table 2:** Long-term outcome of Ramsay Hunt syndrome facial palsy by patient’s self-assessment questionnaire compared to the initial facial palsy grade

Medication at Ramsay Hunt syndrome onset	Number of patients (total = 81)	Medication onset time from Ramsay Hunt diagnose/patient number Initial HB grade^a^ –› patient self-graded facial palsy outcome^b^/patient number			
		≤ 24 h	≤ 48 h	≤ 72 h	> 72 h
Valaciclovir orally 1 g × 3 × 7	10	9 HB II –› 1/1 HB III –› 1/1 HB VI –› 2/1 HB ? –› 1/2 HB ? –› 2/4		1 HB ? –› 1/1	
Valaciclovir orally, dosing unclear	3	3 HB V –›1/1 HB V –› 2/1 HB ? –› 1/1			
Valaciclovir orally 1 g × 3 × 7 + corticosteroid^c^	32	28 HB II –› 1/1 HB III –› 1/2 HB III –› 4/1 HB IV –› 1/2 HB IV –› 2/2 HB V –› 2/1 HB V –› 3/1 HB VI –› 1/1 HB VI –› 3/3 HB ? –› 1/7 HB ? –› 2/5 HB ? –› 3/2	2 HB II –› 1/1 HB V –› 1/1	1 HB V –› 1/1	1 HB V –› 3/1
Aciclovir orally 800 mg × 5 × 7	6	5 HB III –› 2/1 HB IV –› 2/1 HB ? –› 1/3			1 HB VI –› 2/1
Aciclovir orally 800 mg × 5 × 7 + corticosteroid^c^	2	2 HB II –› 2/1 HB ? –› 1/1			
Aciclovir orally, dosing under 800 mg × 5 × 7 or unclear	5	4 HB IV –› 1/1 HB ? –› 1/2 HB ? –›2/1	1 HB ? –› 3/1		
Aciclovir intra venous under 10 mg/kg/day × 3 or unclear	2	2 HB II –› 1/1 HB ?–› 4/1			
Aciclovir orally under 800 mg × 5 × 7 or intra venous under 10 mg/kg/day × 3 + corticosteroid^c^	3	3 HB V –› 2/1 HB II –› 1/1 HB ? –›1/1			
Aciclovir intra venous minimum 10 mg/kg/day × 3	12	10 HB II –›1/1 HB III –›1/1 HB V –› 2/1 HB V –› 3/1 HB VI –› 2/1 HB ? –› 1/2 HB ? –› 2/2 HB ? –› 4/1	1 HB ? –› 1/1		1 HB II –› 3/1
Aciclovir intra venous minimum10mg/kg/d × 3 + corticosteroid^c^	4	3 HB II –› 2/1 HB V –› 1/1 HB VI –› 3/1			1 HB ? –› 3/1
No medication	2				2 HB ? –› 2/1 HB ? –› 3/1

^a^Initial facial palsy grade was available from 40 patient records, graded by House-Brackmann scale (HB) or Sunnybrook scale, latter converted here to HB grades

^b^Facial palsy outcome by patient’s own assessment: 1—totally recovered; 2—slight sequelae; 3—obvious sequelae; 4—severe sequelae

^c^Corticosteroid usually Prednisolon 60 mg/day with tapering dosing 10 mg/day after 5 days for 10 days or Medrol 32 mg or 64 mg/day for 7–10 days

Patients are grouped by the medication type and medication start time at syndrome onset

**Table 3:** Outcome of facial palsy by patient’s self-assessment questionnaire in comparison to initial facial palsy grade with patients (total number = 75) receiving any amount of virus medication (± corticosteroid) within 72 h from Ramsay Hunt syndrome onset

House–Brackman (HB) facial palsy grade at Ramsay Hunt syndrome onset	Totally cured (1): patient number/%	Slight sequelae (2): patient number/%; 1 + 2%	Obvious sequelae: patient number/%	Severe sequelae: patient number/%
HB II	6/75%	2/25%; 100%		
HB III	4/67%	1/16%; 83%		1/16%
HB IV	3/50%	3/50%; 100%		
HB V	4/40%	4/40%; 80%	2/20%	
HB VI	1/14%	2/29%; 43%	4/57%	
HB ?	21/55%	12/32%; 87%	3/8%	2/5%
Total patient number/% per 75 patients	39/52%	24/32%; (1 + 2) 84%	9/12%	3/4%

**Table 4:** Outcome of facial palsy by patient’s self-assessment questionnaire in comparison to initial facial palsy grade with patients (total number 62) receiving maximal virus medication (± corticosteroid) within 72 h from Ramsay Hunt syndrome onset

House–Brackman (HB) facial palsy grade at Ramsay Hunt syndrome onset	Totally cured (1): patient number/%	Slight sequelae (2): patient number/%; 1 + 2%	Obvious sequelae: patient number/%	Severe sequelae: patient number/%
HB II	4/67%	2/33%; 100%		
HB III	4/67%	1/16%; 83%		1/16%
HB IV	2/40%	3/60%; 100%		
HB V	3/43%	2/28%; 71%	2/28%	
HB VI	1/14%	2/29%; 43%	4/57%	
HB ?	17/55%	11/36%; 91%	2/6%	1/3%
Total patient number/% per 62 patients	31/50%	21/34%; (1 + 2) 84%	8/13%	2/3%

The number of patients receiving their medication later than 72 h or taking no medication at all was low, only 6 (7% of 81), but regardless of the initial FP grade, none recovered totally and only 2 (33% of 6) recovered to having only slight sequelae (Table 2).

### FP recovery from patient records and questionnaire combined

Combining questionnaire results and patient chart results, FP outcome could be estimated to 116 (97%) of the 120 patients (Tables 1, 5). Of all the patients, 107 (89%) received virus medication (± corticosteroids) within 72 h and for 103 of them (96%) a long-term FP outcome could be determined; 87 (84%) were cured totally or were left only with slight sequelae (Tables 1, 5). Two patients (2%) took no medications and for 11 (9%) patients the medication was started over 72 h from the RHS onset. FP outcome was known for 12 of these 13 patients: 7 (58%) were cured totally or were left only with slight sequelae; and 5 (42%) were left with obvious or severe sequelae. The patient number in each initial HB grade was as follows: HB II, 3; HB V, 1; HB VI, 2; and HB?, 7. A Fisher’s two-sided Exact Test failed to reach significance (*P* = 0.060) when comparing the results of these two groups: those receiving any medication within 72 h and those receiving medication over 72 h from RHS onset or none at all. Tables 5, 6, 7, and 8 show that there was no significant difference on FP long-term outcome between the different types of medications. Initial total paralysis seamed to lead to worse outcome results in all medication groups (Tables 1, 5, 6, 7, 8).

**Table 5:** Outcome of facial palsy (known for 103 patients) by patient records and patient’s self-assessment questionnaire in comparison to the initial facial palsy grade with patients (total number 107) receiving any amount of virus medication (± corticosteroid) within 72 h from Ramsay Hunt syndrome onset

House–Brackman (HB) facial palsy grade at Ramsay Hunt syndrome onset	Totally cured (1); patient number/%	Slight sequelae (2); patient number/%; 1 + 2%	Obvious sequelae; patient number/%	Severe sequelae; patient number/%	Outcome unknown; patient number
HB II	8/80%	2/20%; 100%			
HB III	4/67%	1/16%; 83%		1/16%	1
HB IV	7/70%	3/30%; 100%			1
HB V	8/50%	5/31%; 81%	3/19%		
HB VI	2/20%	2/20%; 40%	5/50%	1/10%	1
HB ?	27/53%	18/35%; 88%	4/8%	2/4%	1
Total patient number/% per 103 patients	56/54%	31/30%; (1 + 2) 84%	12/12%	4/4%	4 (4% of 107 patients)

**Table 6:** Outcome of facial palsy (known for 86 patients) by patient records and patient’s self-assessment questionnaire in comparison to the initial facial palsy grade with patients (total number 90) receiving maximal amount of virus medication (± corticosteroid) within 72 h from Ramsay Hunt syndrome onset

House–Brackman (HB) facial palsy grade at Ramsay Hunt syndrome onset	Totally cured (1): patient number/%	Slight sequelae (2): patient number/%; 1 + 2%	Obvious sequelae: patient number/%	Severe sequelae: patient number/%	Outcome not known: patient number
HB II	6/75%	2/25%; 100%			
HB III	4/67%	1/ 16%; 83%		1/16%	1
HB IV	5/62%	3/38%; 100%			1
HB V	7/54%	3/23%; 77%	3/23%		
HB VI	2/20%	2/20%; 40%	5/50%	1/10%	1
HB ?	21/51%	16/39%; 90%	3/7%	1/3%	1
Total patient number/% per 86 patients	45/52%	27/31%; (1 + 2) 83%	11/13%	3/4%	4 (4% of 90 patients)

**Table 7:** Outcome of facial palsy (known for 52 patients) by patient records and patient’s self-assessment questionnaire in comparison to initial facial palsy grade with patients (total number 54) receiving any amount of virus medication with corticosteroids within 72 h from Ramsay Hunt syndrome onset

House–Brackman (HB) facial palsy grade at Ramsay Hunt syndrome onset	Totally cured (1): patient number/%	Slight sequelae (2): patient number/%; 1 + 2%	Obvious sequelae: patient number/%	Severe sequelae: patient number/%	Outcome not known; patient number
HB II	3/60%	2/40%; 100%			
HB III	2/67%	0/0%; 67%		1/33%	
HB IV	5/71%	2/29%; 100%			1
HB V	5/50%	3/30%; 80%	2/20%		
HB VI	3/38%	0/0%; 38%	4/50%	1/12%	1
HB ?	9/47%	8/42%; 89%	2/11%		
Total patient number/% per 52 patients	27/52%	15/29%; (1 + 2) 81%	8/15%	2/4%	2 (4% of 54 patients)

**Table 8:** Outcome of facial palsy (known for 51 patients) by patient records and patient’s self-assessment questionnaire in comparison to initial facial palsy grade with patients (total number 53) receiving any amount of virus medication without corticosteroids within 72 h from Ramsay Hunt syndrome onset

House–Brackman (HB) facial palsy grade at Ramsay Hunt syndrome onset	Totally cured (1): patient number/%	Slight sequelae (2): patient number/%; 1 + 2%	Obvious sequelae: patient number/%	Severe sequelae: patient number/%	Outcome not known; patient number
HB II	5/100%				
HB III	2/67%	1/33%; 100%			1
HB IV	2/76%	1/33%; 100%			
HB V	3/50%	2/33%; 83%	1/17%		
HB VI		2/67%; 67%	1/33%		
HB ?	17/55%	10/32%; 87%	2/6%	2/6%	1
Total cure patient number/% per 51 patients	29/57%	16/31%; 1 + 2/88%	4/8%	2/4%	2 (4% of 53 patients)

## Discussion

### FP outcome

In our study, good recovery (total recovery or recovery with only slight sequelae; comparable to HB grades I and II [17]) was obtained by over 80% of the patients receiving antiviral therapy within 72 h from RHS onset. These results are in concordance with results from other studies where medical therapy (antivirals ± corticosteroids) has been used. Even though in our study almost all patients received antiviral therapy, only half received simultaneous corticosteroid treatment. A good recovery rate has been reported by Uri et al. [18] for 26/31 (83%) patients (HB I & II); Murakami et al. [14] for 65/80 (81%) (HB I & II); Shim et al. [1] for 223/328 (68%) (HB I & II); Kiniski [19] for 85/91 (93%); Zainine et al. [20] for 10/15 (67%) (HB I & II); and Coulson et al. [21] for 60/101 (59%) (HB I & II). Before the use of virus or combination medication, worse recovery results were reported. In Peitersen’s study [6], “fair recovery” with either full recovery or only mild sequelae was reported in 46% of cases, and in a study by DeVriese et al. [7] only 30% recovered to HB I & II. Our results, even with antivirals as the only medication, were amongst the best recovery results published. The previous use of acyclovir could also worsen the outcome results compared to the later and present use of valasiclovir with easier dosing regimen. It remains to be seen whether the outcome results continue to improve with the wider usage of newer antivirals (e.g. famciclovir) as suggested in the study by Kim et al. [22].

When comparing the recovery results to the initial HB grade, the low patient numbers in each HB grade made statistically significant conclusions impossible, but there seemed to be a clear drop in the possibility of a favorable outcome if the paralysis was total at the onset. An outcome with only slight sequelae or better was only a little over 40%, whereas already in HB V with any used virus medication it was 70–80%. Still, the outcome seemed better than for those receiving no medication or starting it later than 72 h although the patient number in both these groups was so small that the results were easily affected by any single patient’s outcome.

The mantra we all have probably repeated, that patients with RHS recover worse than those with Bell’s palsy, is still likely to be true, but the gap might not be as wide as we have thought. If we assess Bell’s palsy patients meticulously, the end result, even with corticosteroid treatment, is that about 30% of the patients are left with some degree of sequelae, although mostly slight [23]; not very different from the results achieved in this study.

### Hearing loss

About half of our study patients experienced hearing loss at some point of the illness, with only 20% reporting constant hearing loss after the illness. Since this result was based on patients’ own reports, other reasons for hearing loss (e.g. prespyacusis) could also influence the outcome. In the study by Coulson et al. [21], 42% of the patients reported hearing loss and 50% had hearing deterioration on the affected site on audiometric measurement (any asymmetry to the unaffected side). Shin et al. [24] reported hearing loss in 74% of the patients studied (≥ 10 dB difference to the unaffected side). Neither of these retrospective studies commented on the end result of the hearing loss. In Peitersen’s prospective FP study [6], 73% of the RHS patients had hearing loss. He states as a rule that the hearing loss is non-reversible. This was not true in our study, even amongst those patients that underwent end-outcome audiometry tests—many recovered. The same view was presented by Wayman et al. [25], with only 5% of the patients with residual hearing loss after RHS.

### Blisters

In our study, herpes blisters were most often found in the ear, followed by the mouth cavity, but also to a minor extent without ear or mouth involvement in other parts of the head and neck. In Peitersen’s study [6], the ear was affected in 66% of cases, face in 15%, and mouth cavity in 9%. In a study by Coulson et al. [21], inclusion criteria for the study consisted of blisters only in the ear or mouth, with the ear affected in 85% of the cases, mouth in 7%, and both in 8% of the cases.

With our patients, the herpes blisters preceded the FP in 48% of the cases, were simultaneous in 16%, and followed the FP in 36% of the cases. In Peitersen’s study [6], blisters occurred first in 60% of the cases, were simultaneous in 25%, and followed FP in 15% of the cases. Aizawa et al. [3] found that blisters preceded the palsy 31%, occurred at the same time as the palsy 52%, and followed the palsy 15%. Hato et al. [26] found a difference between children and adults in the way that blisters followed FP in 32% of adults, but even in 50% of children 16 years or younger. In our study, over one third of the RHS diagnoses could not be made at FP onset because the blisters were yet to come. It is a serious reminder to advise the patients to be alert of blisters appearing after FP. It is very probable that some of these patients are lost for diagnosis and treatment in cases of blisters following FP.

In our study design, we set the time boundaries for the herpes blisters to be 1 month before or after the FP and some patients showed the blisters 14–30 days before or after FP. In the study by Aizawa et al. [3], the blisters were seen from 27 days before to 13 days after the FP. They studied the viral load and immune responses of the patients and concluded that FP in RHS can occur at different times, from early phase to the regression phase of VZV reactivation, and that there are variable patterns of development of facial nerve dysfunction induced by VZV reactivation and the progression of neuritis.

## Strengths and limitations

The strength of this study was the rare long-term outcome assessment of FP by self-assessment of a rather large group of patients. The exploration of many different characteristics of RHS was also valuable, revealing, among other things, the knowledge that with over one third of the patients, the blisters appeared after FP onset and in 20% of the patients the blisters were hidden in the ear canal or in the mouth, and could not be seen if not looked for.

A limitation of the study was that the protocol failed to permit standardized gradings of FP from the patient records at the baseline visit or at the end of the follow-up period. Facial function evaluations varied from brief mentions of “almost total paralysis”, “slight weakness”, “almost cured”, or “well cured” to the use of both HB and SB grading scales. The study data was collected already several years ago and goes back over 20 years. We hope that our routines on evaluating FP patients are more coordinated today than what they were in the 1990s, and that the SB grading scale is frequently used also by residents who usually first meet these patients in the outpatient clinics. In addition, the time the patient was first seen and evaluated after FP onset and the length of the follow-up varied between patients. The time gap from RHS onset to answering the questionnaire was also long in some cases, which likely affected how the patient was able to remember and answer about the symptoms at the disease onset. Finally, the number of patients in subgroups was also too small to draw statistically liable conclusions.

## Conclusions

Medication seems to significantly advance the outcome of RHS, thus it is important to recognize the disease. In our study, only 16% of patients displayed blisters occuring simultaneously with FP, so it is crucial to ask the patients about preceding blisters and inform them about the possibility of blisters occurring after FP. In addition, 18% of our patients experienced their blisters at the ear canal or in the mouth, hidden from view, therefore it is imperative to meticulously check the patient throughout.
